# The Eco-Immunological Relevance of the Anti-Oxidant Response in Invasive Molluscs

**DOI:** 10.3390/antiox12061266

**Published:** 2023-06-13

**Authors:** Davide Malagoli, Nicola Franchi, Sandro Sacchi

**Affiliations:** 1 Department of Life Sciences, University of Modena and Reggio Emilia, 41125 Modena, Italy; nicola.franchi@unimore.it (N.F.); sandro.sacchi@unimore.it (S.S.); 2 NBFC, National Biodiversity Future Center, 90133 Palermo, Italy

**Keywords:** ecoimmunology, stress, immunity, haemocytes, biodiversity, *Pomacea canaliculata*, *Achatina fulica*, *Mytilus galloprovincialis*, *Dreissena polymorpha*, alien species

## Abstract

Reactive oxygen species (ROS) are volatile and short-lived molecules playing important roles in several physiological functions, including immunity and physiological adaptation to unsuitable environmental conditions. In an eco-immunological view, the energetic costs associated with an advantageous metabolic apparatus able to cope with wide changes in environmental parameters, e.g., temperature range, water salinity or drought, could be further balanced by the advantages that this apparatus may also represent in other situations, e.g., during the immune response. This review provides an overview of molluscs included in the IUCN list of the worst invasive species, highlighting how their relevant capacity to manage ROS production during physiologically challenging situations can also be advantageously employed during the immune response. Current evidence suggests that a relevant capacity to buffer ROS action and their damaging consequences is advantageous in the face of both environmental and immunological challenges, and this may represent a trait for potential invasiveness. This should be considered in order to obtain or update information when investigating the potential of the invasiveness of emerging alien species, and also in view of ongoing climate changes.

## 1. Introduction

Climate change and invasive species are among the most relevant threats to biodiversity [1]. Invasive alien species are demonstrating an outstanding capacity to adapt to the new environment they colonize. Beside the adaptations to new physical-chemical environmental aspects, invasive species must also present a high degree of metabolic plasticity and an outstanding capacity of adaptation to new antigenic ecospaces [2,3]. In the IUCN list of the worst invasive species [4], numerous invertebrate species are retrievable, presenting hypervariable defence-related molecules and specific anti-microbial peptides [5,6]. In addition to the immune features and mediators that evolved or differentiated along specific evolutionary lineages, one widespread function of the immune anti-pathogen responses is represented by the controlled production of reactive oxygen species (ROS) [7,8]. These volatile and short-lived molecules are of extreme importance both in invertebrate and vertebrate immune response and, thanks to their non-specificity, they can be directed against a plethora of potential pathogens. Beside this, environmental conditions can also create oxidative stress in organisms [1,9]. In this last case, the ROS become a menace for the organism itself, and oxidative stress must be managed in order to avoid permanent damage.

Oxidative stress is a ubiquitous phenomenon that can be studied at cellular, tissue, organ and organism level. It occurs when the production or presence of ROS exceeds antioxidant defence capabilities [10,11]. ROS-mediated oxidative stress can be promoted either from the environment [12,13] or from the activity of endogenous enzymes, such as those involved in cellular respiration or various forms of oxydase [14]. The main cellular effects of oxidative stress are unspecific and include lipid peroxidation, protein misfolding and nucleotide alteration [15] although other more complex and less investigated effects cannot be excluded [16].

As cellular respiration and metabolism are natural and constitutive sources of ROS [14], all cells have evolved mechanisms of defence that include enzymes, e.g., catalases, peroxidases and superoxide dismutase and molecular scavengers, e.g., glutathione. More complex molecular chaperones, such as heat shock proteins (HSPs), can also intervene in recovering the protein misfolding promoted by oxidative stress [17]. While the molecular bases of oxidative stress and their association with the immune defences are well conserved, differences exist among organisms in the ability to manage ROS production during the immune response [18] and physiologically challenging situations such as those experienced as a consequence of a sudden change in oxygen availability, e.g., changes in water level or arousal after estivation/hibernation periods [12,17,19,20].

## 2. The Oxidative Stress Response against Immune or Environmental Challenges in Highly Invasive Mollusc Species

The resistance to oxidative stress introduces a further level of complexity in the proper management of invasive species. It has already been documented that on some occasions the usage of biocides may positively affect the spread of invasive species. An experiment performed by exposing native and invasive ant species to sublethal doses of a neonicotinoid, a class of highly diffused chemical biocides, demonstrated that it could either increase or decrease the probability of invasive ant survival according to the exposure status of the native ants [21]. Neonicotinoids may have important effects in non-target organisms. These effects include DNA damage, protein oxidation and lipid peroxidation that are largely the consequence of the increased ROS concentration that follows the altered mitochondrial Ca^2+^ homeostasis and the hindered mitochondrial respiration [22]. The invasive species endowed with a high resistance to ROS-mediated insults are less likely to suffer consequences from those pesticides provoking ROS-mediated oxidative stress; either the invasive species are the direct target of the treatment or they are non-target species. This further restricts the number of the compounds available for pest control, pushing researchers to investigate the effects of biological control [23] or biopesticides [24]. However, as is described in more detail below, biopesticide effects can also be limited by highly efficient ROS detoxification.

The 2013 IUCN list of the worst invasive species [4] includes bivalves, e.g., *Mytilus galloprovincialis*, *Dreissena polymorpha* and *Potamocorbula amurensis* and gastropods, e.g., *Achatina fulica*, *Euglandina rosea* and *Pomacea canaliculata*. Although a different level of information is available for these species, the retrievable data point towards a common trait of an efficient immune response and a relevant resistance to oxidative stress.

The Mediterranean mussel *Mytilus galloprovincialis* presents circulating haemocytes (immunocytes) that support efficient cell-mediated innate immune functions [25,26,27,28]. ROS species have been demonstrated to intervene during immune response and after the tissue injury of *M. galloprovincialis* [29,30]. As a filter feeder and a species of economic relevance, several studies have investigated the effects of environmental pollutants on Mediterranean mussel health and anti-oxidant stress response [13,30]. As the increase in water temperature may represent a threat for mussel cultivation, the possibility to improve *M. galloprovincialis* heat stress response has been studied at physiological and molecular levels. It has been demonstrated that a brief pre-exposure to thermal stress (a procedure labelled as “hardening”) significantly increases the capability of mussels to cope with a further and more prolonged exposure to high temperatures [31]. Among the numerous parameters taken into account, oxidative stress and redox signalling were studied by measuring the expression of different superoxide dismutases, glutathione-S-transferase and catalase, the enzymatic activities of superoxide dismutases, catalase and glutathione reductase and the protein levels of HSP70 isoforms (i.e., HSP72 and HSP73). Superoxide dismutase and catalase expression during the prolonged exposure to high temperatures were increased, and they were significantly higher in hardened mussels with respect to stressed but non-hardened animals. For some molecules, the increase in enzymatic activities followed a similar temporal profile in hardened and non-hardened mussels, but it was higher in hardened ones. Glutathione reductase acted similarly to superoxide dismutases and catalase. Consistently, HSP72 and HSP73 protein levels were higher in hardened animals, and the increased expression of electron transport system elements seemed synchronized with that of the other components of the anti-oxidant response [31]. The complex and adaptive anti-oxidant response of hardened mussels to the experimental thermal stresses suggested that hardened mussels are able to manage and coordinate both the metabolic and the oxidative stress responses in the face of sudden temperature increase.

The zebra mussel *D. polymorpha* is an invasive species that can also be studied as a sentinel organism in eco-physiological studies. As frequently reported in molluscs [32], the haemocytes can be divided into two main morphologies, namely agranular and granular haemocytes. Agranular haemocytes may include cells with different morphologies and, possibly, functions, i.e., blast-like cells and hyalinocytes. In the zebra mussel, the latter exhibited the highest oxidative activity, also in comparison with granular haemocytes [33]. In ex vivo experiments, *D. polymorpha* haemocytes exposed to chemical, physical and biological stresses elicited a complex response that included the expression of anti-oxidant enzymes and they changed significantly on the basis of the stressor considered [34]. Numerous studies included the effects of cadmium, as the zebra mussel has been proposed as a sentinel organism in freshwater environments for the evaluation of water pollutant effects [35]. The oxidative activity of *D. polymorha* haemocytes changed in different haemocyte sub-populations, as the granular cells were the less affected by the treatment and the hyalinocytes seemed to be influenced only at the highest doses of cadmium ions, indicating an important stability of mitochondrial activity and ROS management for these two cell populations [36], even in the presence of relevant stresses.

To our knowledge, no information comparable to that reported for *M. galloprovincialis* and *D. polymorpha* is available for the Asian clam *P. amurensis*. This notwithstanding, experiments correlating the HSP protein levels to water salinity values, as well as studies comparing the aerobic-fermentative metabolism rates in relation to water salinity and seasonal temperature changes, have been presented [37,38,39], suggesting that the Asian clam could also finely adjust stress response and metabolism as a consequence of environmental changes.

**Figure 1:**
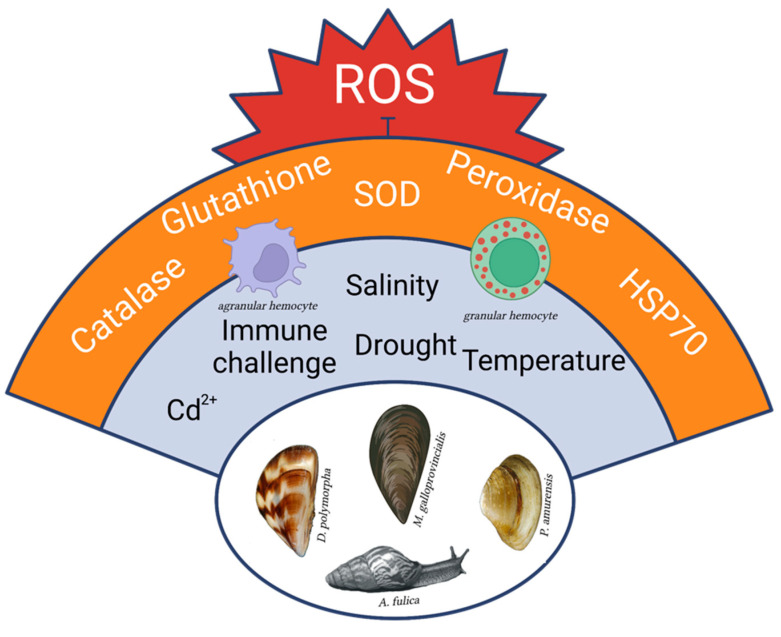
Highly invasive molluscs of different Classes present common traits of resistance versus oxidative stress that could be originated either from environmental stimuli or from immune challenges. The advantage of managing ROS increase is shared among diverse physiological responses, thus limiting the associated energy expenditure and allowing efficient energy trade-offs [47]. Created with BioRender.com (accessed on 2 May 2023).

The African giant snail *A. fulica* has been labelled as the most widely distributed invasive pest land snail [40]. Its large haemocytes were described a long time ago; they displayed phagocytic activity, were able to synthesise superoxide anion radical, but did not present endogenous peroxidase activity [41]. As the intermediate vector of the human pathogenic nematodes of the genus *Angiostrongylus*, the immune response of *A. fulica* to these parasites has been investigated. In snails infected with *Angiostrongylus vasorum* larvae, increased phenoloxidase (PO) activity and consequently a ROS-mediated immune response were observed, with a PO activity peak in the immediacy of the infection. The activation of PO was also followed by melanisation. Beside PO activation, the immediate increase in nitric oxide (NO) production was also observed via Griess reaction, confirming that the immune response against the nematode included a significant ROS-mediated component, especially during the first phases of the infection [42]. Ex vivo experiments performed by confronting withdrawn haemocytes with the axenic larvae of diverse metastrongyloid lungworms also demonstrated that *A. fulica* haemocytes can release their nuclear content, thus forming extracellular traps (ETs). Beside containing histones that exert a well-known antimicrobial function [43], the *A. fulica* ETs also contained molecules similar to myeloperoxidase, an enzyme involved in the production of hypohalous acids and exerting a cytotoxic function especially by means of oxidative stress [44]. The exposure of *A. fulica* to the causative agent of human eosinophilic encephalitis, *Angiostrongylus cantonensis*, promoted the metabolic shift towards oxidative activity by increasing the glycolytic pathway and the activity of lactate dehydrogenase in order to keep the redox balance, at least in the haemolymph [45]. As an invasive snail, *A. fulica* has also been the subject of experiments aimed at controlling its diffusion without damaging other species. In this regard, the efficacy of a nematode, *Phasmarhabditis hermaphrodita*, also used as a component of a bio-based molluscicide, has been assessed on the giant African snail. Juvenile snails are highly resistant to the nematodes as they are able to entrap them into the inner layer of the shell, apparently without further metabolic or immune responses [46] (Figure 1).

While no specific information is retrievable about the immune defences and oxidative responses of the rosy wolfsnail that, however, can be vehicle of *A. cantonesis* [48], more data can be retrieved for the snail *P. canaliculata*. The circulating haemocytes of *P. canaliculata* have been described [49,50,51] and their proteome is available [52]. In addition to the haemolymph, the haemocytes have also been recognized within organs and in regenerating tissues [53,54], increasing the number of potential sites where the haemocyte-mediated immune response can take place and haemocytes can replicate. The immune system of *P. canaliculata* has been the target of numerous studies focused on the control of its spread. The vegetal pesticide pedunsaponin A modified the haemocyte number, membrane potential and morphology and promoted ciliary loss in ciliated tissues, thus affecting snail respiration and excretion [55,56]. The effects of the pesticide were increased after silencing the expression of HSP70 by RNAi [57], suggesting a role for this chaperone in buffering pedunsaponin A-mediated damage. As already reported above for *A. fulica*, the immune system of *P. canaliculata* was also targeted by using a commercially available molluscicide based on the nematode *P. hermaphrodita*. *P. canaliculata* exhibited a significant resistance to this molluscicide. The recommended concentration of *P. hermaphrodita* determined an overall low mortality and reduced the synthesis of an antimicrobial peptide alternatively in two immune-related organs of the snails, i.e., the gills and the anterior kidney, in dependence of the temperature of the treatment [58]. The ultrastructural observation and proteomic analysis of an organ associated with the oxidative stress resistance and potentially involved in the immune response, namely the aortic ampulla [59,60], revealed that *P. canaliculata* systemic reaction to the immune challenge, represented by the pathogenic nematode, involved a diffused oxidative stress response, as suggested by the increased expression in the ampulla of enzymes associated with ROS detoxification, such as Cu^2+^–Zn^2+^ superoxide dismutase, catalase-like isoform X1, glutathione peroxidase-like protein and peroxiredoxin. As the nematodes were not present in the ampulla of exposed snails, and no nematode proteins were isolated or sequenced among the ampulla proteome, the increase in anti-oxidant enzymes allowed the speculation that the organ was responding to a systemic increase in ROS. Consistently, the snail self-protection from ROS-mediated damage was also associated with the increased expression of HSP60, 70 and 90 [61].

## 3. The Eco-Immunological Advantage of Managing Oxidative Stress Response

While significant differences exist in the anatomical organization and physiology of the invasive species introduced above, their high tolerance versus oxidative stress represents a common trait in their immune response towards pathogens, pesticides and environmental or experimental physico-chemical stressors. The invasiveness of a species relies on different physiological features [62,63], not least the capacity to overcome or tolerate the new potential pathogens. Invertebrate innate-only immune response has been progressively described as anticipatory and highly specific, especially in light of the numerous hypervariable molecules discovered in invertebrate taxa [64,65,66,67,68] and the increasing evidence of circulating microbiotas [69,70,71] that some invertebrate species are able to manage together with the intestinal microbiota. However, a specific and anticipatory immune response could also miss new pathogens that may be encountered during the colonization of new environments by the invasive species. In this respect, well-conserved and unspecific responses, such as the increased synthesis of ROS, could become essential for the adaptation to new environments. As an unspecific response based on short-lived and volatile molecules, ROS-mediated immune defence is self-limited by the capability of the host to manage the potential harm that these volatile molecules could determine; in the majority of the invasive species mentioned here, a marked resilience versus oxidative stress has been reported.

**Figure 2:**
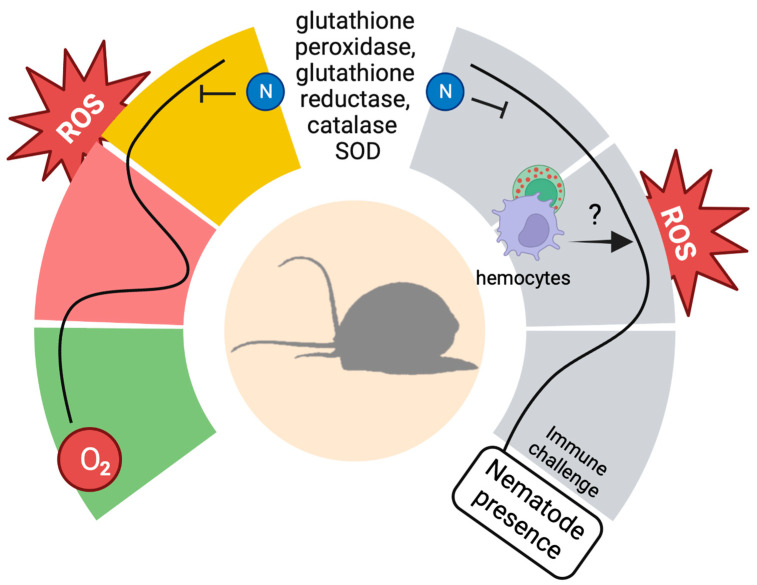
The invasive gastropods *A. fulica* and *P. canaliculata* can cope with ROS peaks in different situations. Left: when the water and oxygen availability are sufficient (green), the snails do not display a significant activity in detoxifying enzymes. When the water availability is reduced, e.g., as a consequence of drought, the snails can enter estivation, reducing their metabolism, as a consequence of reduced oxygen availability (red). Once the environmental conditions become more suitable for their survival, the snails face a peak of ROS that follows the increase in the oxygen availability and in the metabolic activity. This peak is associated with an increased enzymatic activity and, at least in *P. canaliculata*, is sustained by nitrogen-containing compounds. Right: during the immune response against pathogenic nematodes (grey), a similar trend of ROS production is observed, although in this case it is also possible that immune-related components, e.g., haemocytes, can contribute to ROS synthesis and increase. The protective elements employed during the arousal can also be utilized in response to the immune challenge, allowing the snails to express a powerful ROS-mediated immune response, avoiding self-damage. Created with BioRender.com (accessed 2 May 2023).

The mussel *M. galloprovincialis* can populate subtidal and intertidal environments. It displays tolerance to temperature fluctuations [72] and its metabolic plasticity allows the Mediterranean mussel to adapt and to react differently to environmental challenges on the basis of the colonized habitat [73]. In *D. polymorpha*, an exceptional tolerance to variable environmental conditions associated with strong anti-oxidant defences has been reported [74], maybe in consequence of a high genetic diversity [75]. *P. amurensis* can colonize subtidal and intertidal habitats and present a high tolerance to wide ranges of water salinity and temperature [76,77], conditions that are connected to changes in oxygen availability and require efficient oxidative stress response. Among invasive freshwater gastropods, both *A. fulica* and *P. canaliculata* can undergo estivation [78,79], a physiological adaptative response to unsuitable environmental conditions that relies on the ability to manage the significant increase in ROS during the arousal, when oxygen concentration quickly increases. In the African giant snail, mitochondrial or cytosolic superoxide dismutase levels did not change during estivation, and the same held true for other anti-oxidant enzymes such as glutathione peroxidase, glutathione reductase or catalase. While their amount and activity are stable during estivation, during the arousal from estivation the antioxidant enzymes increase their detoxifying activities with a time- and tissue-specific distribution. Consistently, no signs of protein damage or lipid peroxidation were detected in the heart tissues of African giant snails after four weeks of experimental estivation [78]. During estivation, *A. fulica* can accumulate urea, a nitrogen waste product less harmful than ammonia. Evidence collected in *A. fulica* has demonstrated an astonishing capacity to detoxify exogenous and injected ammonia via a significant increase in the urea synthesis rate, suggesting that urea could have other metabolic roles beside the excretory one [80]. In *P. canaliculata*, the involvement of nitrogen compounds in the anti-oxidant response associated with the arousal from estivation has been documented in detail. In agreement with the observations from *A. fulica*, the *P. canaliculata* presented a complex pattern of anti-oxidant responses that involved enzymatic and non-enzymatic components which fluctuated in an organ-specific fashion, especially during the arousal [81]. The nitrogen compound uric acid is likely to play an important role in buffering the ROS concentration increase during the arousal [82]. A specific tissue, labelled as urate tissue, is distributed among numerous organs, i.e., the lung, the digestive system, the anterior kidney (also identified as pallial ureter) and the ampulla, which are considered relevant organs in managing ROS levels during the arousal [60,79] (Figure 2).

Recently, the response of *P. canaliculata* circulating haemocytes has also been investigated, and differences have emerged in the mediators involved in the anti-oxidant response versus experimental hibernation or estivation [83], confirming the high plasticity and adaptability of the oxidative stress response in this invasive snail. The proteomic analysis of ampullae collected from snails challenged with the pathogenic nematode *P. hermaphrodita* evidenced a metabolic response that paralleled the adaptation to estivation, with a significant increase in anti-oxidant defences and an increase in aerobic metabolism, witnessed by the increase in mitochondrial enzymes related to ATP synthesis [61].

The ability to cope with the environmental conditions that require an increased capability of managing oxidative stress response represents a significant advantage that could help the adaptivity and invasiveness of some species. Nonetheless, that capacity is associated with the metabolic costs that the maintenance of a prompt anti-oxidant defence system requires. In the eco-immunological perspective of efficient energy expenditure and management [84,85], the advantage of controlling the self-damaging consequences of oxidative stress response could be more relevant if extended to other physiological functions such as the immune response against pathogens. While the physiological immune functions are deeply interconnected with metabolism, neural functions and development [86], and their energetic demand is balanced together with that of the other physiological systems, during the response against pathogens the energy demand of the immune system suddenly increases and must be managed [84]. In this context, the possibility to take advantage of a pre-set and dynamic anti-oxidant defence system such as those described above for invasive molluscs may allow the organisms to express a potent and efficacious ROS-mediated immune response without incurring the self-damage that ROS may determine for the host. In organisms adapted to sustain the oxidant stress response derived from changes in water temperature or salinity, or the arousal from estivation or hibernation, the self-limitations that must be applied to the ROS-mediated immune response [87] could be less marked. This would prove particularly functional for *A. fulica* and *P. canaliculata* in the presence of large pathogens, such as the nematode *P. hermaphrodita*. From this perspective, the metabolic costs associated with the adaptation to specific environmental conditions could be counterbalanced by the advantages also reflecting on the immune functions, thus giving the species endowed with this capacity a special advantage in the face of both environmental and immunological challenges [1].

## 4. Conclusions

The updated determination of alien species’ potential of invasiveness and the consequent polices for alien species control are becoming more urgent in view of the increasing consequences of ongoing climate change. For instance, in 2012, the EU Parliament classified *Pomacea* as a pest and invading genus and requested a comprehensive risk assessment on *Pomacea* species [88] that included the potential establishment of these snails in EU territory. Similar studies highlighted regions of India considered as more prone to invasion by the African giant snail, *A. fulica* [89]. The different scenarios regarding the areas potentially invaded and prospected in those risk assessments almost ten years ago, which considered specific patterns of air, water temperature and rainfall fluctuations, might now need some adjustments or updates. At the same time, climate change has also promoted an unanticipated reduction of sea water pH. This acidification has been demonstrated to modify the freeze tolerance of subtidal species, such as for instance *M. galloprovincialis*, and it will likely modify the area of distribution of subtidal and intertidal *Mytilus* species [90].

Current evidence suggests that most invasive molluscs present a relevant capacity to manage ROS increase, either derived from environmental cues or from immune stimuli. From an eco-immunological perspective, the possibility of taking advantage of the same metabolic ability in diverse functional contexts allows a better use of resources and energy trade-offs. This shared capacity may likely represent a trait of potential invasiveness which, associated with more species-specific characteristics, could justify the success of species such as *M. galloprovincialis*, *D. polymorpha, A. fulica* and *P. canaliculata* to invade and adapt to new environments and niches. From this perspective, the resistance to experimental oxidative stress of emerging alien species could be further investigated in order to gain more complete information on their potential of invasiveness and to formulate consistent policies for their control.

## Data Availability

No new data were created or analysed in this study. Data sharing is not applicable to this article.
